# Effects of mindfulness-based music listening on conflict control in young adults with insomnia disorder: behavioral and event-related potential evidence

**DOI:** 10.3389/fpsyg.2024.1404000

**Published:** 2024-09-09

**Authors:** Huijuan Shi, Yi Liu, Yong Liu, Maoping Zheng, Xiaolin Liu

**Affiliations:** ^1^Chongqing Institute of Foreign Studies, Chongqing, China; ^2^School of Psychology, Southwest University, Chongqing, China; ^3^School of Music, Southwest University, Chongqing, China; ^4^Mental Health Institute of Chinese Music, Southwest University, Chongqing, China

**Keywords:** mindfulness-based music listening, emotion–cognition interaction, conflict control, hyperarousal, ERPs

## Abstract

**Introduction:**

Insomnia Disorder (ID) has become the second most prevalent mental disorder, with significant negative effects on daytime cognitive functions. Previous studies suggested that mindfulness-based music listening (MBML) can effectively improve conflict control and attentional processing in healthy adults. However, the behavioral and neurophysiological characteristics of MBML in young adults with ID remain unclear.

**Methods:**

To explore the behavioral and neurophysiological characteristics of MBML in regulating negative emotions among young Chinese adults with ID, 60 young adults with ID were asked to complete an emotion-word Stroop task under three mood states while recording event-related potentials (ERPs).

**Results:**

Task and questionnaire results showed that (1) negative emotion induced by the negative simulated video significantly suppressed the attentional processing of emotional faces and words in the conflict control task among young people with ID, (2) MBML reduced cognitive and physical arousal levels, enhanced positive mood, and improved attentional control abilities in young adults with ID. The ERP results showed that a greater N3 effect and the smaller P3 and late positive component (LPC) effects reflected that MBML effectively regulated negative emotions induced by the negative simulated video and attentional processing abilities for conflict control in young adults with ID.

**Discussion:**

Maintaining mindfulness while listening to music may enhance positive emotional experiences and improve cognitive ability, and exhibit larger N3 effects and smaller P3 and LPC effects in the electrophysiology mechanism, with a reduction in the hyperarousal level in young adults with insomnia disorders.

## 1 Introduction

Insomnia disorder (ID) seriously threatens individuals’ physical and mental health (Ding et al., 2024; Zhao et al., 2021). ID has become the second most prevalent mental disorder (Wardle-Pinkston et al., 2019; Zhao et al., 2021), Cao et al. (2017) conducted a meta-analysis on the incidence rate of insomnia in the general population of China in 2017 and found that the prevalence of insomnia disorders in China was approximately 15%, and younger Chinese adults suffer from more insomnia compared to older adults. ID is defined as a persistent difficulty in initiating and/or maintaining sleep and/or waking up earlier than desired (Zhao et al., 2021). ID is currently the most common sleep disorder characterized by difficulty falling asleep, difficulty maintaining sleep, and early awakening, accompanied by impaired daytime cognitive functions, such as alertness, memory, attention, and executive function across different populations worldwide (Schneider et al., 2019; Zhao et al., 2018). Previous studies have shown that long-term insomnia has negative effects on physical and mental health, such as depression and anxiety in the general population of China (Cao et al., 2017; Zhao et al., 2018), and daytime cognitive functions, such as attention and executive function in the population worldwide (Wardle-Pinkston et al., 2019; Zhao et al., 2021).

### 1.1 Hyperarousal for insomnia disorders and music therapy

Recently, much etiological and pathophysiological evidence has shown that hyperarousal is “a state of relatively increased arousal in physiological, cortical, and cognitive-emotional domain” (Dressle and Riemann, 2023). In insomnia disorders, hyperarousal includes three functions: (1) as a predisposing factor, (2) as a state marker for insomnia, and (3) as a pathophysiological component that potentially leads to or causes various insomnia-related symptoms. Hyperarousal during insomnia often leads to cognitive dysfunctions, such as conflict control (Ding et al., 2023) and cognitive flexibility (Ding et al., 2024; Uddin, 2021), and triggers negative emotions such as anxiety and depression (Ding et al., 2024; Dopheide, 2020). As a tool for measuring the level of arousal in insomnia disorders, the Pre-Sleep Arousal Scale (PSAS) includes two dimensions of cognitive pre-sleep arousal and physical pre-sleep arousal (Nicassio et al., 1985) and effectively assesses the sleep quality of individuals with insomnia disorders (Gong et al., 2016; Schneider et al., 2019). In recent years, based on the hyperarousal model for insomnia disorders, researchers have conducted relevant studies on insomnia disorders using integrated intervention strategies (Gong et al., 2016; Schneider et al., 2019).

With the burgeoning interest in using music to treat insomnia (Chang et al., 2012; Dickson and Schubert, 2019; Luo et al., 2024; Majeed et al., 2021), studies have shown that various methods of music therapy, such as passive listening therapy and music performance therapy, can effectively alleviate symptoms associated with insomnia (Chang et al., 2012; Lai et al., 2014) and regulate negative emotions of mental disorders (Dickson and Schubert, 2019; Huang et al., 2017; Lai et al., 2014). With the in-depth exploration by more music therapy researchers, the methods and strategies of music therapy are constantly being optimized and updated (Lai et al., 2014; Umemoto et al., 2021; Zhu et al., 2023). Aesthetics-based music listening, as an effective intervention method, has been proven to be the most widely used in music therapy (Liu et al., 2021b,c; Liu et al., 2023; Susino, 2023). The integration of music therapy with other psychological therapies is becoming a new trend and is being widely applied (Miranda, 2021; Susino, 2023; Umemoto et al., 2021; Zhu et al., 2023). It is worth noting that musical works using the treatment for insomnia have characteristics such as smoothness, slowness, and relaxation (Chang et al., 2012; Lai et al., 2014; Luo et al., 2024), which perfectly align with the requirements of mindfulness meditation training (Liu et al., 2021a).

### 1.2 MBML intervention and conflict control for insomnia disorders

Mindfulness meditation, as an economical, safe, and effective intervention strategy, has been widely used to enhance the psychological wellbeing of healthy individuals (Hernandez-Ruiz and Dvorak, 2020; Liu et al., 2021a) and provide the psychological treatment for patients with mental disorders, such as depression, anxiety, and insomnia (Dopheide, 2020; Gong et al., 2016; Rusch et al., 2018; Tomaselli, 2014; Wittmann and Schmidt, 2014). Previous studies have shown that mindfulness meditation “can serve as an auxiliary treatment to medication for sleep complaints” and may mildly improve cognitive dysfunction, regulate anxiety, and alleviate depression in individuals with insomnia disorders (Dressle and Riemann, 2023; Dopheide, 2020; Gong et al., 2016). Mindfulness meditation has also been integrated with other therapies, such as cognitive behavioral therapy and family therapy (Smith et al., 2019), resulting in positive and promising clinical effects (Edinger et al., 2021; Ziegler et al., 2019).

Recent research has found that mindfulness-based music listening (MBML), as an effective intervention strategy in music therapy, has a significant regulatory effect on negative emotions (Liu et al., 2021a,c). MBML intervention strategies primarily focus on the behavioral performance of individual attention processing and different emotional states under individual emotional–cognitive interaction (Liu et al., 2021a,c; Wardle-Pinkston et al., 2019). Conflict control is “the ability of the brain to monitor conflicts in the process of information processing and measures the ability of inhibitory control at the cognitive level” (Liu et al., 2021a). Conflict control in emotion–cognition interactions (Raschle et al., 2017) effectively reveals the improvement effect of mindfulness and music listening on individual physical and mental health and cognitive function (Liu et al., 2021c).

Previous studies have shown that the integration of Questionnaire-Stroop Task-ERP technology (Liu et al., 2021a,c) may provide a practice approach for measuring the arousal level of individuals with insomnia disorders in physiological, cortical, and cognitive-emotional domains (Dressle and Riemann, 2023; Ding et al., 2023; Ding et al., 2024). Previous studies have shown that the emotional face-word Stroop task is an effective paradigm for detecting emotion–cognition interactions and attentional control processing under different emotional states (Liu et al., 2021a). Under consistent or inconsistent conditions of Stroop trials, task accuracy (ACC) and reaction times (RTs) reflect the behavioral and neural correlates of conflict control in different emotional states (Liu et al., 2021a; Xue et al., 2015).

### 1.3 Electrophysiological mechanisms of conflict control in insomnia disorders

From the perspective of emotion–cognition integration (Dennis, 2010), event-related potentials (ERPs) effectively reveal individual behavior and ERP correlates of emotion–cognition interaction, providing an effective measure for evaluating different emotional states and cognitive functions in the emotional face-word Stroop task (Raschle et al., 2017; Weth et al., 2015). Previous research has indicated that emotions significantly impact individual attention processing and cognitive function (Raschle et al., 2017), and different emotional states affect individual conflict control (Liu et al., 2021a). Different ERP indicators can effectively evaluate the behavior and neural correlation in both healthy and unhealthy individuals (Liu et al., 2020a,b, 2021c). Previous studies found, compared to healthy sleepers, individuals with insomnia disorders exhibited lower accuracy, slower reaction times, and lower N450 amplitude in the color-word Stroop task, along with a larger P3 amplitude in the two-choice oddball task (Ding et al., 2023). In addition, studies have shown that the left dorsolateral prefrontal cortex plays an important role in conflict control for individuals with insomnia disorders (Ding et al., 2024).

Previous studies have explored emotional–cognitive interactions using conflict control tasks and found that the N3, P3, and LPC of EEG indicators reflect attentional processing and emotional regulation in conflict control (Liu et al., 2021a). In research on attentional processing and emotional regulation, N3 is a useful variable for exploring the emotion–cognition processing of visual stimuli, characterized by a negative amplitude occurring approximately 250–350 ms after the stimulus onset (Truman and Mudrik, 2018). N3 is associated with semantic violations in conflict control, and the N3 effect reflects attentional processing in the recognition of the target stimulus and the classification of semantic matching or mismatch (Maguire et al., 2013). Previous studies have found that a larger N3 effect occurs during the initial classification of targets in a neutral mood state (Draschkow et al., 2018; Truman and Mudrik, 2018).

P3 is associated with higher cognitive resources and is a positive component that occurs approximately 300–600 ms after the appearance of a stimulus. In emotion–cognition interactions, the P3 effect is an effective EEG indicator reflecting positive or negative emotional states in emotion–cognition interactions (Liu et al., 2020a,b, 2021a). Larger P3 amplitudes reflect the consumption of more cognitive resources in negative emotional states, while smaller P3 amplitudes indicate the consumption of fewer cognitive resources in calm emotional states (Ding et al., 2023; Liu et al., 2021a; Yuan et al., 2007). The late positive component (LPC) is a key indicator for evaluating emotional–cognitive interaction effects (Liu et al., 2021c; Mengfan et al., 2020). As a positive component, the LPC exists approximately 600–1,000 ms after stimulus onset. Previous studies have found that negative emotions induce a greater LPC effect than neutral or positive emotions (Liu et al., 2020a,b, 2021a).

In summary, this study was based on the hyperarousal model of insomnia disorder theory (Dressle and Riemann, 2023; Riemann et al., 2010), using the emotional face-word Stroop task (Liu et al., 2021a) and a combination of questionnaire-behavioral performance-EEG technology to explore the behavioral and neural correlates of MBML intervention strategies on emotional regulation and conflict control in young Chinese adults with ID. The within-participant (pre-test, mid-test, and post-test in emotional face-word Stroop task) and between-participant (mindfulness-based music listening group, MMG; the wait-list control group, WCG) differences in the conflict control were examined to illustrate the effect of different emotional states evoked by music on conflict control in young adults with ID. The purpose of this study was to explore the effects of MBML on cognitive function in Chinese young adults with insomnia disorders. Based on previous studies, we hypothesize the following:

- First, MBML intervention may reduce the arousal level of young adults with insomnia disorders, manifested as lower scores for MMG in the post-test compared to the pre-test; on the post-test of PSAS, the scores for MMG are lower than those for WCG in cognitive and physical pre-sleep arousal level, with no difference between MMG and WCG in the pre-test of PSAS.
- Second, there will be no significant intergroup differences between MMG and WCG in PANAS score, TMS or MAAS score, task performance (response time and accuracy), and ERPs (P3 amplitude and LPC amplitude) in the pre-test phase (baseline).
- Third, compared with the baseline of the pre-test phase, the negative emotions of young adults with insomnia disorders induced by sad music will be manifested as lower PA scores and higher NA scores in the mid-test phase and lower ACC and slower RTs in the task performance; consuming more cognitive resources on ERPs, this will be manifested as larger N3 amplitude, smaller P3 amplitude, and larger LPC amplitude, with no between-group differences.
- Fourth, compared with the negative emotion induction of the mid-test phase, MBML would induce a mindfulness state in young adults with insomnia disorders, manifested as higher TMS or MAAS scores for MMG than those for WCG; in the task performance, this will be manifested as higher ACC and faster RTs for MMG than those for WCG; consuming fewer cognitive resources on ERPs, this will be manifested as smaller N3 amplitude, larger P3 amplitude, and smaller LPC amplitude for MMG than those for WCG.

## 2 Methods

### 2.1 Participants

Three hundred college students with insomnia disorders (56.5% female students, M_age_ = 21.53, SD_age_ = 1.72) were recruited through campus advertising and evaluated for the severity of their insomnia disorder using the Pre-Sleep Arousal Scale (PSAS) (Nicassio et al., 1985). Then, sixty college students with the highest PSAS scores (58.3% female students, M_age_ = 20.92, SD_age_ = 1.09) were included in this study and assessed their sleep arousal levels (Riemann et al., 2010) using the PSAS (Nicassio et al., 1985). According to the study requirements, participants were randomly divided into two groups: the mindfulness-based music listening group (MMG, *N* = 30) and the wait-list control group (WCG, *N* = 30). They were required to avoid taking substances or drugs that affect attention. All participants did not take any psychotropic drugs for 7 days before the experiment and reported normal hearing and speech, normal or corrected-to-normal vision, and no other psychological disorders, except insomnia. Before the experiment began, all participants were required to read the experimental instructions and provide informed consent. This study was approved by the Southwest University Ethics Committee (IRB No. H22117).

### 2.2 Stimuli

#### 2.2.1 Musical stimuli

Two calm music pieces were selected as music stimuli for emotion regulation, based on the purpose of this study and existing research results on emotion regulation (Liu et al., 2021a; Luo et al., 2024; Mizrahi Lakan et al., 2023). In addition, our previous research results (Liu et al., 2021c) have shown that calming music can induce more positive emotional experiences, such as calmness, nostalgia, warmth, and joy (Liu et al., 2021a,c). To evaluate the potential impact of familiarity on emotional regulation, we asked participants to report their familiarity with the musical stimuli. Participants reported that they were unfamiliar with the two calm musical pieces. The two calm music pieces were selected from a stimulus set consisting of six complete Chinese classical folk instruments (Liu et al., 2021c), and the duration of the two calm music pieces was approximately 6 min. In the current study, the smoothness, slowness, and relaxation of music stimuli were evaluated by all participants, and Cronbach’s alpha for music stimuli was 0.83.

#### 2.2.2 Experimental simulation video

The simulated video was derived from a real Chinese event of COVID-19 in 2020 with a duration of 8 min, and was used to induce negative emotions in all participants in the current study (Liu et al., 2021a). The video stimulation’s emotional valence was negative, such as sadness and tension.

#### 2.2.3 Mindfulness meditation audio

The Chinese version of the mindfulness meditation audio (Liu et al., 2021c) was selected from Liu et al. (2021a). The duration of the audio recording was 10 min and was recorded in MP3 format.

### 2.3 Questionnaire

#### 2.3.1 The positive and negative affect schedule

The Positive and Negative Affect Schedule (PANAS) (Watson and Clark, 1988) evaluated participants’ positive and negative emotional trajectories induced by music. The PANAS is a negative and positive affect questionnaire that includes 20 items of emotional adjectives (Chin and Rickard, 2013) that describe current feelings on a 5-point scale ranging from 1 (*very slightly or not at all*) to 5 (*extremely*). The total scores for positive and negative affect are summed separately. In the current study, the PANAS had Cronbach’s alpha of 0.79 and was used to assess participants’ mood states before and after the experiment.

#### 2.3.2 The pre-sleep arousal scale

This study used the Pre-Sleep Arousal Scale (PSAS) to assess an individual’s sleep arousal levels (Nicassio et al., 1985). It includes 40 items, and PSAS is widely used to measure cognitive awakening and physical awakening among adults with ID (Schneider et al., 2019). For each item, the participants’ responses ranged from 1 (not at all) to 5 (very much); the higher the total score, the higher the level of sleep arousal, indicating poorer sleep quality. Cronbach’s alpha of the PSAS was 0. 81.

#### 2.3.3 The Toronto mindfulness scale

The Toronto Mindfulness Scale (TMS) is widely used to evaluate an individual’s mindfulness state and could effectively predict intervention effects (Chung and Zhang, 2014; Lau et al., 2006). The Chinese version of the TMS (Chung and Zhang, 2014) contains 13 items and uses a 5-point scale ranging from 0 (*not at all*) to 4 (*very much*), and the higher the scores, the higher the mindfulness state (Ireland et al., 2019; Lau et al., 2006). In this study, TMS had Cronbach’s alpha of 0.70 and was used to assess participants’ mindfulness before and after the experiment.

#### 2.3.4 The mindful attention awareness scale

The MAAS is a questionnaire used to measure the mindfulness state or the level of awareness of individual mindfulness traits (Carlson and Brown, 2005; Deng et al., 2012). The Chinese version of the MAAS was revised by Si-yi et al. (2012) and Deng et al. (2012). It has 15 items. Participants are required to report their actual feelings in the last week on a 6-point scale ranging from 1 “almost always” to 6 “almost never.” The higher the score, the higher the mindfulness state or the level of awareness and attention in daily life. In this study, the MAAS was used to measure and evaluate participants’ mindfulness state and had Cronbach’s alpha of 0.88.

### 2.4 The emotional face-word Stroop task

**Figure 1:**
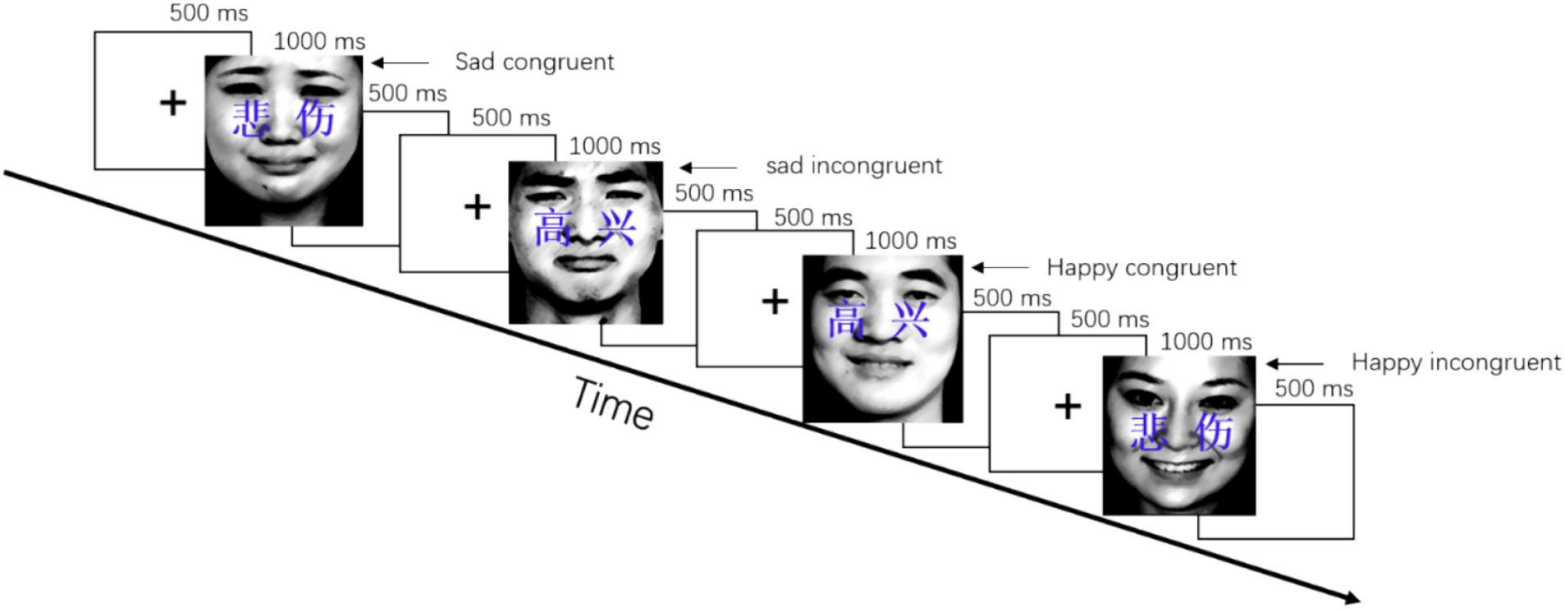
Example of the experimental task. The stimuli were completely randomized. The Chinese characters on the face read happy (高兴) and sad (悲伤).

In this study, the revised emotional face-word Stroop task (Liu et al., 2021a) was used to explore the effects of conflict control and attentional processing on different affective states among young adults with ID. The visual stimuli of the experimental task consisted of 40 face images (10 sad faces for male students and 10 sad faces for female students; 10 happy faces for male students and 10 happy faces for female students) [extracting from the Chinese Affective Picture System (Wang and Luo, 2005)] and emotional words (sad and happy), forming four stimulus conditions: sad congruency, sad incongruency, happy congruency, and happy incongruency (Carretié et al., 1997; Liu et al., 2019a, 2021a; Truman and Mudrik, 2018). In the Stroop task (Figure 1), the stimulus for 1,000 ms was presented after a fixation point for 500 ms, followed by a blank screen interval for 500 ms. In the experimental task, the participants were asked to press the buttons as quickly as possible, with pressing “1” button for congruent conditions and pressing “2” buttons for incongruent conditions. The stimuli were randomly presented.

To assess facial and semantic processing of young adults with ID, respectively, the experimental task was divided into two sub-tasks of emotional faces and emotional words. The stimuli were randomly presented and repeated twice and formed 160 trial experimental sub-tasks. The two sub-task orders were counterbalanced across the participants. There is an exercise module of 80 trials before the Stroop task of 320 trials. Each image was identical in size (300 × 260 pixels), resolution (96 dots per inch), brightness, and background. The image stimuli are presented in the center of a 21-inch computer display screen. In EEG data collection, the participant was asked to avoid eye blinking as much as possible during the completion of Stroop tasks to reduce experimental artifacts.

### 2.5 Procedure

The current study adopted a double-blind design, which recruited experimental assistants for collecting experimental data, and college students with insomnia disorders who participated in the experiment. In this study, the experimental data consist of three parts: questionnaire data, task data, and ERP data. To minimize researcher bias, questionnaire data, task data, and ERP data were analyzed by different individuals. Participants were randomly assigned to the MMG or the WCG group. The experiment was divided into three sections: the pre-test phase (the baseline measurement), the mid-test phase (negative emotion induction), and the post-test phase (the mindfulness-based music listening intervention). All participants were required to complete the PANAS, PSAS, TMS, and MAAS scales, as well as the Stroop task (pre-test) prior to the task to access their baseline level. In the negative emotion induction phase, all participants were asked to watch an 8-min sad movie clip, rate their emotional states using the PANAS again, and complete the Stroop task (the mid-test). During the mindfulness-based music listening intervention phase, the MMG received 10 min of mindfulness meditation training and listened to calm music for 5 min, while the WCG sat still for 15 min. All participants completed the PANAS, TMS, MAAS, and PSAS and finally completed the Stroop task (post-test). The EEG data were recorded throughout the experiment. The entire experiment lasted approximately 1 h.

### 2.6 Study design and data analysis

Independent sample t-tests were used to evaluate the between-group differences in age and sex. Within- and between-group differences in PANAS, PSAS, TMS, and MAAS scores were evaluated using repeated-measures analysis of variance (ANOVA). A repeated-measures ANOVA [3 (test: Pre-test, mid-test, post-test) × 2 (group: MMG; WCG) × 4 (condition: Sad congruency, sad incongruency, happy congruency, and happy incongruency)] was used for the ACC and RTs of emotional faces and emotional words in the experimental task, with task and mood state as within-participant factors and group as between-participant factors. SPSS (version 28.0) was used to analyze the experimental data. The multiple comparisons of *post-hoc t*-tests were adjusted using Bonferroni, while the *p*-value for sphericity was adjusted using the Greenhouse–Geisser method.

### 2.7 The EEG recording and analyses

A tin electrode mounted on an elastic cap from 32 scalp sites (Neuroscan, Charlotte, NC, United States) was used to record brain electrical activity, with the fronto-central aspect (REF) as the reference electrode and the medial frontal aspect (GRD) as ground electrode. An electrode placed infraorbital near the left eye was used to record vertical electrooculogram, and all inter-electrode impedances were maintained below 5 kΩ. MATLAB R2023a was used to process ERP data.

Based on the individual’s grand ERP averages of correct trials, we create the emotional face and word stimuli. The data from 256 to 1,000 Hz were sampled, and high- and low-pass filtering at 0.1 Hz and 45 Hz was performed, respectively, with selecting the left and right mastoids as the reference sites. Data were epoched from 300 ms prior to stimulus onset to 1,000 ms after the presentation and were baseline-corrected to the pre-stimulus interval. Trials were excluded if they included electrooculogram (EOG) artifacts (ocular movements and eye blinks); artifacts owing to amplifier clipping, bursts of electromyographic activity, or peak-to-peak deflections exceeding ±80 μV also were excluded from averaging before independent component analysis (ICA).

No differences in trial counts of between- or within-group for the emotional faces and emotional words sub-tasks were found. Moreover, components of EOG artifacts and head movements in the ICA results were removed after visual inspection. Based on previous studies and emotion–cognition interactions of the topographical distribution of the grand-averaged ERP activities (Liu et al., 2020a,b, 2021a), their time epochs were selected for analysis: Three time windows from 250 to 350 ms [N3 component, (Liu et al., 2021a; Maguire et al., 2013)] and 300 to 600 ms [P3 component, (Liu et al., 2020a,b; Madsen et al., 2019)] and a late time window from 600 to 1,000 ms [late positive component: LPC, (Liu et al., 2019b, 2021a)]. These ERP component latencies were assessed relative to the onset of the visual stimulus, which included four conditions in the emotional faces and emotional words sub-tasks (sad congruency, sad incongruency, happy congruency, and happy incongruency).

The following regions of interest (ROIs) (see Liu et al., 2021c) were selected: frontal (F3, Fz, F4), frontal–central (FC3, FCz, FC4), central (C3, Cz, C4), central–parietal (CP3, CPz, CP4), parietal (P3, Pz, P4), and occipital (O1, OZ, O2) regions. For the conflict control task, repeated measures of ANCOVA [3 (test: post-test, mid-test, post-test) × 4 (condition: sad congruency, sad incongruency, happy congruency, and happy incongruency) × 2 (group: MMG, WCG) × 6 (electrode: frontal, frontal–central, central, central–parietal, parietal, and occipital sites)] were conducted on the amplitudes of N3, P3, and LPC, with group as a between-participant factor and task, condition, and electrode as within-participant factors. SPSS (version 28.0) was used to analyze the data. The multiple comparisons of *post-hoc t*-tests were adjusted using Bonferroni, while the *p*-value for sphericity was adjusted using the Greenhouse–Geisser method.

## 3 Results

### 3.1 Questionnaire results

**Figure 2:**
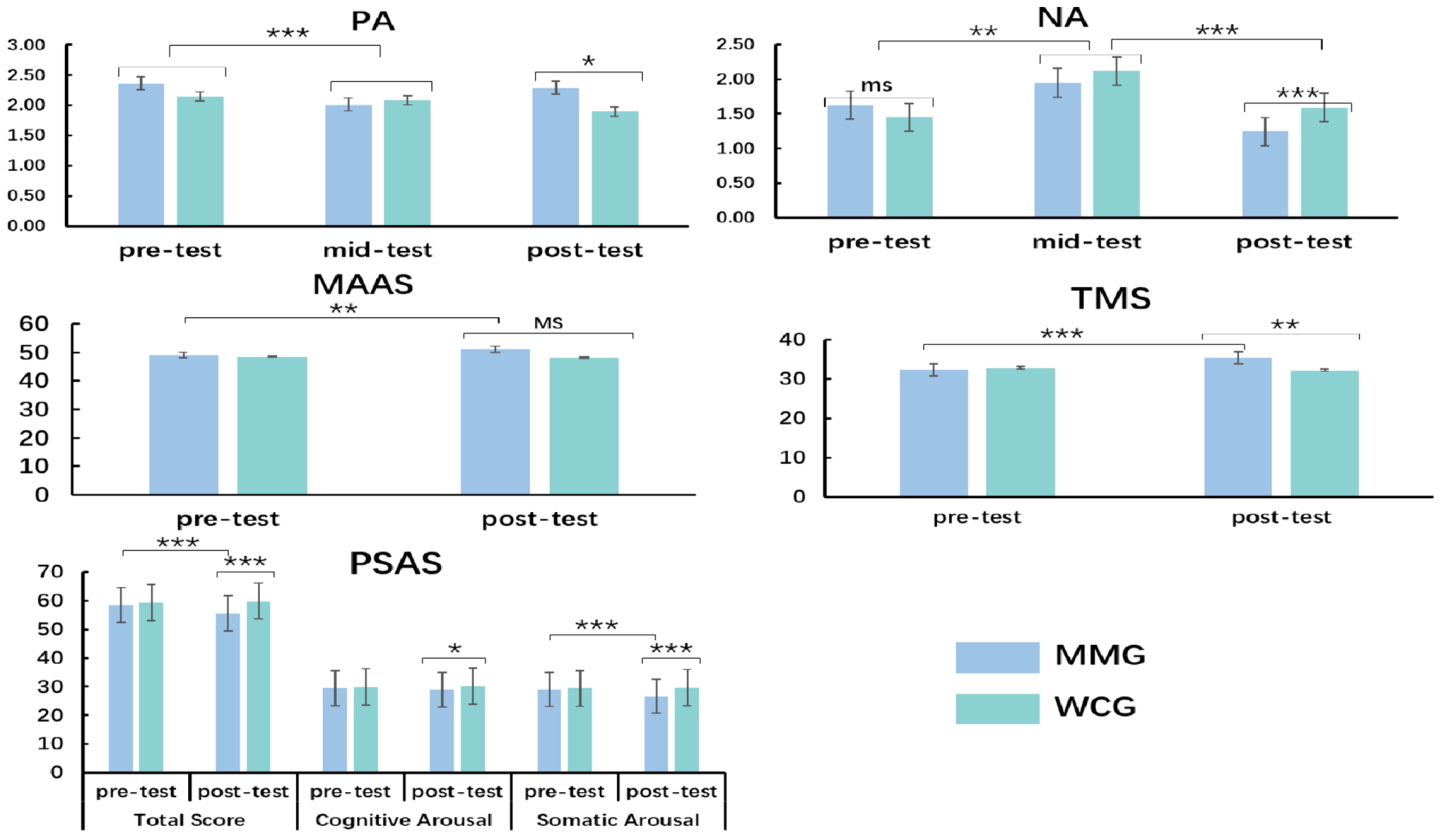
Scale results of between group differences in different test phases. PA, positive affect; NA, negative affect; PSAS, Pre-Sleep Arousal Scale; TMS, Toronto Mindfulness Scale; MAAS, *Mindful Attention Awareness Scale*; MMG, mindfulness-based music listening group; WCG, wait-list control group; ms, significant marginally difference; *p<0.05, **p<0.01, and ***p<0.001.

Participants’ demographic information and questionnaire results are presented in Table 1 and Figure 2. No significant between-group differences were observed in age or sex (all *ps* > 0.05).

**Table 1:** Participants’ demographic information and questionnaire results.

Variables			MMG (M ± SD)		WCG (M ± SD)		*t*
			*n* = 30		*n* = 30		
Age			21.03	0.96	20.8	1.22	0.83
Sex			Male =12, Female =18		Male =13, Female =17		
PANAS	PA	Pre-test	2.36	0.09	2.15	0.08	0.78
		Mid-test	2.01	0.12	2.08	0.11	0.29
		Post-test	2.28	0.13	1.90	0.14	0.81
	NA	Pre-test	1.53	0.05	1.45	0.06	0.59
		Mid-test	1.95	0.11	2.12	0.12	0.59
		Post-test	1.25	0.07	1.59	0.06	0.93
PSAS	CA	Pre-test	29.50	1.68	30.00	2.20	0.13
		Post-test	29.00	1.20	30.27	1.91	0.37
	SA	Pre-test	29.03	0.35	29.33	0.35	0.39
		Post-test	26.67	0.39	29.63	0.39	0.97
TMS		Pre-test	32.37	4.25	32.83	4.16	0.05
		Post-test	35.57	5.04	32.27	3.16	0.37
MAAS		Pre-test	49.10	4.94	48.57	5.68	0.85
		Post-test	51.13	4.24	48.07	8.22	0.23

PANAS, Positive and Negative Affect Schedule; PA, positive affect; NA, negative affect; PSAS, Pre-Sleep Arousal Scale; CA, cognitive arousal; SA, somatic arousal; TMS, Toronto Mindfulness Scale; MAAS, Mindful Attention Awareness Scale; MMG, mindfulness-based music listening group; WCG, wait-list control group; M, mean; SD, standard deviation.

Repeated-measures ANOVA on PANAS scores (Figure 2) indicated that there was no main effect of the group (*p* > 0.05). A main effect of the test was recorded (*F* (2, 57) = 37.35, *p* < 0.001, *η*2 *p* = 0.39), and the *post-hoc t*-test showed that compared to the pre- and post-test, the NA score was highest and the PA score was lowest in the mid-test. In the post-test phase, compared to WCG, MMG has higher PA scores and lower NA scores, there were no between-group differences in the pre- and mid-test. The PANAS results indicated that the simulated video significantly induced all participants’ negative emotions (ranked in descending order of scores: Upset >distressed>scared >terrifying) in the mid-test phase, and MBML significantly improves negative emotions in young adults with ID in the post-test.

There was a main effect of the test on PSAS scores (Figure 2) (*F* (1, 58) = 21.42, *p* < 0.001, *η*2 *p* = 0.27), and the post-test score was lower than the pre-test. A main effect of the group was found (*F* (1, 58) = 13.54, *p* < 0.001, *η*2 *p* = 0.19), and the scores for MMG were lower than those for WCG. The result of PSAS suggested that MBML reduced cognitive and physical arousal levels in young adults with ID, particularly with a more significant improvement in somatic arousal.

There was a main effect of the test on TMS scores (Figure 2) (*F* (1, 58) = 7.43, *p* = 0.008, *η*2 *p* = 0.11), and the *post-hoc t*-test found that the TMS scores for MMG were higher than that of WCG in the post-test, with no between-group difference in pre-test (*p* > 0.05). From Cronbach’s alpha of TMS (0.70), there is a problem of low validity in the structural validity of the Chinese version of TMS (Chung and Zhang, 2014; Ireland et al., 2019). To make up for this limitation, we mainly used the results of MAAS to evaluate the mindfulness state of participants induced by MBML.

Repeated-measures ANOVA on MAAS scores found no main effect of test and group (all *p* > 0.05), there was an interaction between test and group, the *post-hoc* t-test showed that the post-test was higher than the pre-test for the MMG score (*p* = 0.005), and a score of the MMG was higher than those of the WCG in the post-test (*p* = 0.074); no insignificant difference between the pre- and post-test for the WCG was found in MAAS (*p* > 0.05). The result of MAAS indicated that mindfulness-based music intervention improved the levels of mindfulness state among the MMG.

### 3.2 Task results

The experimental task in this study consisted of two sub-tasks: The Emotional Faces Sub-task for detecting facial recognition and the Emotional Words Sub-task for evaluating semantic processing.

#### 3.2.1 The emotional faces sub-task

**Figure 3:**
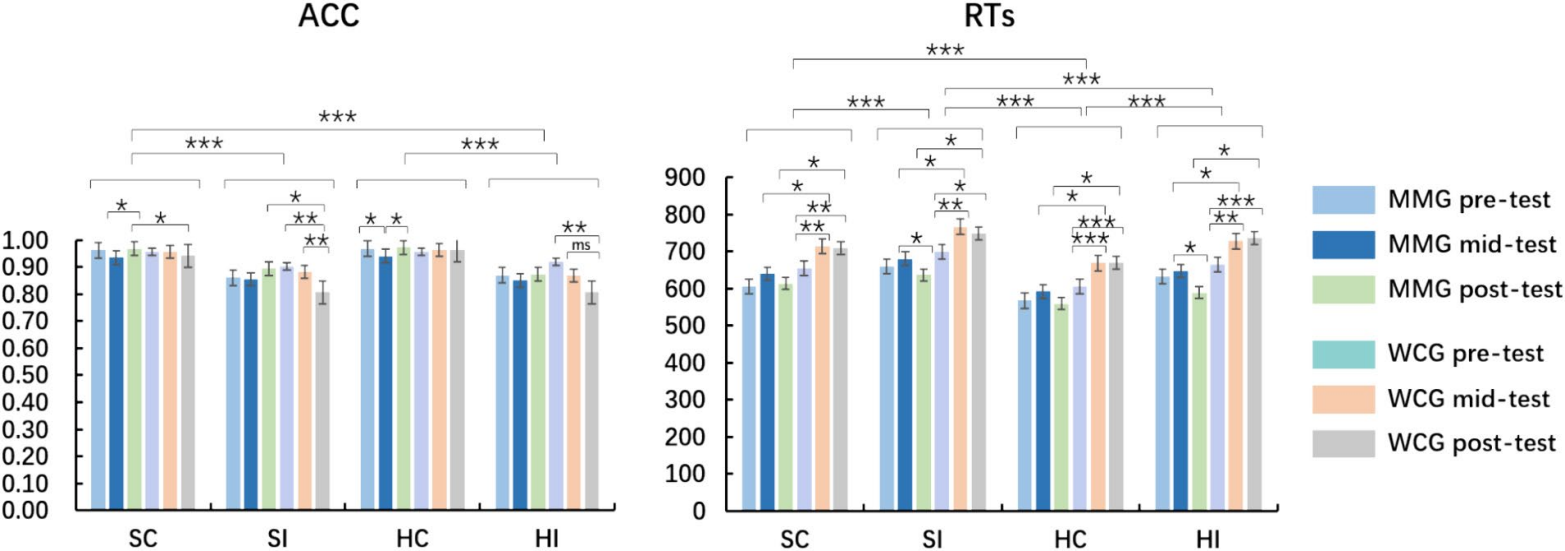
Accuracy (ACC) and reaction time (RT) within- and between-group differences in the emotional faces sub-task; MMG, mindfulness-based music listening group; WCG, wait-list control group; SC, sad congruency; SI, sad incongruency; HC, happy congruency; HI, happy incongruency; ms, significant marginally difference; *p < 0.05, **p < 0.01, and ***p < 0.001.

The results of the participants’ emotional faces for the three mood states are provided in Table 2 and Figure 3.

**Table 2:** Descriptive statistics on accuracy and reaction times of emotional feces in three mood states.

Variables		MMG (M ± SD)						WCG (M ± SD)					
		*n* = 30						*n* = 30					
		Pre-test		Mid-test		Post-test		Pre-test		Mid-test		Post-test	
ACC	SC	0.96	0.01	0.94	0.01	0.97	0.01	0.96	0.01	0.96	0.01	0.94	0.01
	SI	0.86	0.02	0.86	0.03	0.90	0.03	0.90	0.02	0.88	0.03	0.81	0.03
	HC	0.97	0.01	0.94	0.01	0.97	0.01	0.96	0.01	0.96	0.01	0.96	0.01
	HI	0.87	0.03	0.85	0.03	0.87	0.03	0.92	0.03	0.87	0.03	0.81	0.03
RTs	SC	606.044	21.459	640.602	23.414	614.122	20.324	655.765	21.459	714.056	23.414	710.073	20.324
	SI	660.473	24.057	680.82	23.861	637.26	21.647	700.204	24.057	767.04	23.861	748.006	21.647
	HC	567.658	19.13	592.779	21.791	559.704	20.136	606.3	19.13	668.876	21.791	669.955	20.136
	HI	633.986	23.17	647.53	25.473	589.521	23.962	664.918	23.17	728.288	25.473	735.958	23.962

ACC, accuracy; RTs, reaction times; SC, sad congruency; SI, sad incongruency; HC, happy congruency; HI, happy incongruency; MMG, mindfulness-based music listening group; WCG, wait-list control group; M, mean; SD, standard deviation.

Repeated-measures ANOVA on the ACC of emotional faces suggested that the main effect of the test was marginally significant (*F* (2, 58) = 2.56, *p* = 0.08, *η*2 *p* = 0.04), and the *post-hoc t*-test found that the ACC of pre-test was higher than that of mid- and post-test (*p* < 0.05), with no significant difference between the mid- and post-test (*p* > 0.05). A main effect of the condition (*F* (3, 57) = 34.75, *p* < 0.001, *η*2 *p* = 0.38) was found, and the ACC of the sad and happy incongruency was greater than those of the sad and happy congruency (*p* < 0.01), with no significant difference within conditions of sad and happy congruency or incongruency (*p* > 0.05). There was no main effect of the group (*p* > 0.05). The interaction effect of the test and group in the ACC of emotional faces was observed (*F* (1, 57) = 7.89 *p* < 0.001, *η*2 *p* = 0.12), and simple effect analysis found that the ACC for MMG was higher than that for WCG in the post-test (*p* = 0.033), with no between-group differences in the pre- and mid-test (*p* > 0.05). An interaction between the test and condition was shown (*F* (2, 56) = 2.55, *p* = 0.02, *η*2 p = 0.04), and the ACC of the sad and happy incongruency was greater than those of the sad and happy congruency (*p* < 0.01), with no difference within conditions of sad and happy congruency or incongruency (all *p* > 0.05). There was no interaction effect of the condition and group (all *ps* > 0.05). There was a triple interaction effect between the test, condition, and group (*F* (1, 57) = 3.65, *p* = 0.002, *η*2 *p* = 0.06), and the ACC for MMG was higher than that for WCG under the conditions of sad congruency or incongruency conditions in the post-test (*p* < 0.05), with no significant between-group differences in the four conditions of the pre- and mid-tests (all *ps* > 0.05).

There was the main effect of the group on RTs of emotional faces (*F* (1, 58) = 8.29, *p* = 0.006, *η*2 *p* = 0.13), and RTs for MMG were faster than that for WCG. A main effect of the test (*F* (2, 57) = 8.55, *p* < 0.001, *η*2 p = 0.13) was found, and RTs of the mid-test were slower than those of the pre-test (*p* < 0.001), with no significant differences between the pre- and post-test and the mid- and post-test (*p* > 0.05). The main effect of the condition was significant (*F* (3, 57) = 64.34, *p* < 0.0001, *η*2 *p* = 0.053), and RTs of sad incongruency were slower than those of the other three conditions, with ratings of RTs in sad incongruency > happy incongruency > sad congruency > happy congruency. An interaction between the test and group was significant (*F* (2, 57) = 6.67, *p* = 0.002, *η*2 *p* = 0.10), and RTs for MMG were faster than those for the WCG in the mid- and post-test (*p* < 0.01); there was no significant between-group difference in the pre-test (*p* > 0.05). No interaction between the condition and group or between the test and condition was recorded (all *ps* > 0.05). The triple interaction effect of the test, condition, and group was not observed (all *ps* > 0.05).

#### 3.2.2 The emotional words sub-task

**Figure 4:**
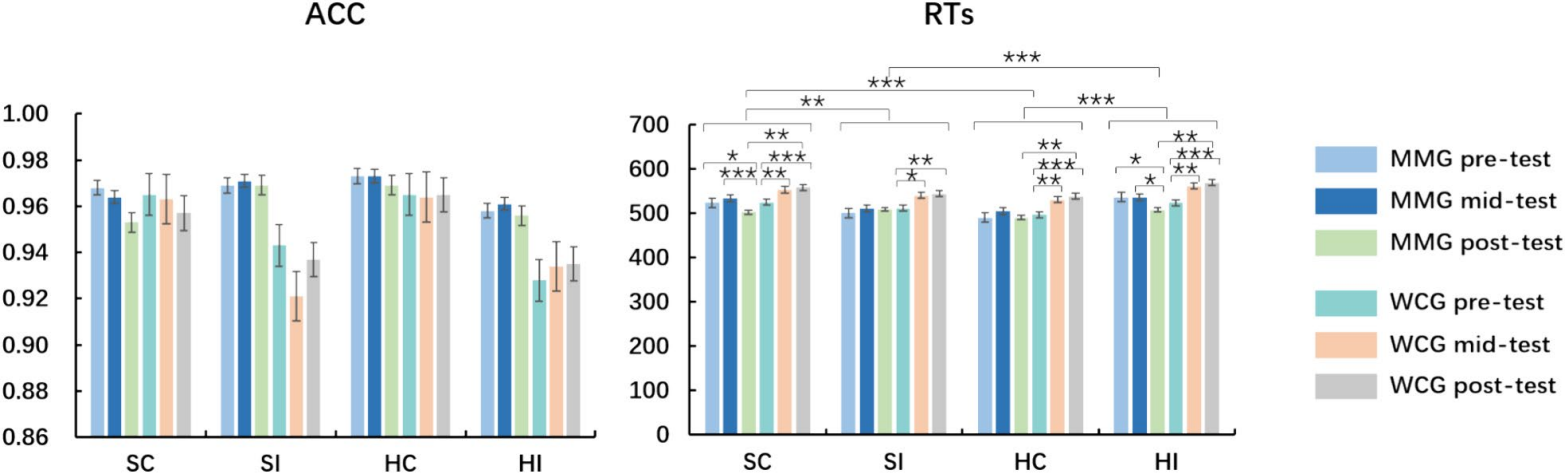
Accuracy (ACC) and reaction times (RTs) within- and between-group differences in the emotional words sub-task; MMG, mindfulness-based music listening group; WCG, wait-list control group; SC, sad congruency; SI, sad incongruency; HC, happy congruency, HI, happy incongruency; *p < 0.05, **p < 0.01, and ***p < 0.001.

The results of the participants’ emotional words for the three mood states are presented in Table 3 and Figure 4.

**Table 3:** Descriptive statistics on accuracy and reaction times of emotional words in three mood states.

Variables		MMG (M ± SD)						WCG (M ± SD)					
		*n* = 30						*n* = 30					
		Pre-test		Mid-test		Post-test		Pre-test		Mid-test		Post-test	
ACC	SC	0.97	0.01	0.96	0.01	0.95	0.01	0.97	0.01	0.96	0.01	0.96	0.01
	SI	0.97	0.02	0.97	0.03	0.97	0.02	0.94	0.02	0.92	0.03	0.94	0.02
	HC	0.97	0.01	0.97	0.01	0.97	0.01	0.97	0.01	0.96	0.01	0.97	0.01
	HI	0.96	0.02	0.96	0.02	0.96	0.02	0.93	0.02	0.93	0.02	0.94	0.02
RTs	SC	522.934	13.056	533.406	13.394	502.171	13.595	524.291	13.056	552.585	13.394	557.854	13.595
	SI	500.519	11.933	511.057	14.09	508.814	16.192	511.032	11.933	539.472	14.09	543.323	16.192
	HC	489.584	11.278	504.542	14.584	489.818	12.198	496.622	11.278	530.193	14.584	537.994	12.198
	HI	535.9	14.151	535.235	15.773	507.23	13.769	523.116	14.151	560.762	15.773	568.344	13.769

ACC, accuracy; RTs, reaction times; SC, sad congruency; SI, sad incongruency; HC, happy congruency; HI, happy incongruency; MMG, mindfulness-based music listening group; WCG, wait-list control group; M, mean; SD, standard deviation.

Repeated-measures ANOVA on the ACC of the emotional words suggested that the main effect of the test, condition, and group was not significant, with no interaction and triple interaction between the test, condition, and group (all *ps* > 0.05).

No main effect of the group on the RTs of emotional words was found. There was a main effect of the test on RTs of emotional words (*F* (2, 57) = 7.16, *p* = 0.001, *η*2 *p* = 0.11), and the RTs of the pre-test were faster than those of the mid- and post-test in emotional words, with no significant difference in the mid- and post-test. There was a main effect of the condition (*F* (3, 57) = 23.89, *p* < 0.001, *η*2 *p* = 0.29), and the RTs of sad incongruency were slower than those of the other three conditions, with ratings of RTs in sad incongruency > sad congruency > happy incongruency > happy congruency.

The interaction between the test and group was recorded (*F* (2, 58) = 9.62, *p* < 0.001, *η*2 *p* = 0.14). Simple effect analysis suggested that the RTs of the MMG were faster than those of the WCG in the post-test (*p* = 0.009), with no significant between-group differences in the pre- and mid-tests. No interaction between the condition and group or test was found. Triple interaction between the test, condition, and group was recorded (*F* (1, 58) = 9.62, *p* < 0.001, *η*2 p = 0.14), and in addition to being happy incongruency, the RTs of the MMG were faster than those of the MMG on other three conditions in the post-test, with no between-group differences in the pre- and mid-test (all *ps* > 0.05).

### 3.3 The ERP results

**Figure 5:**
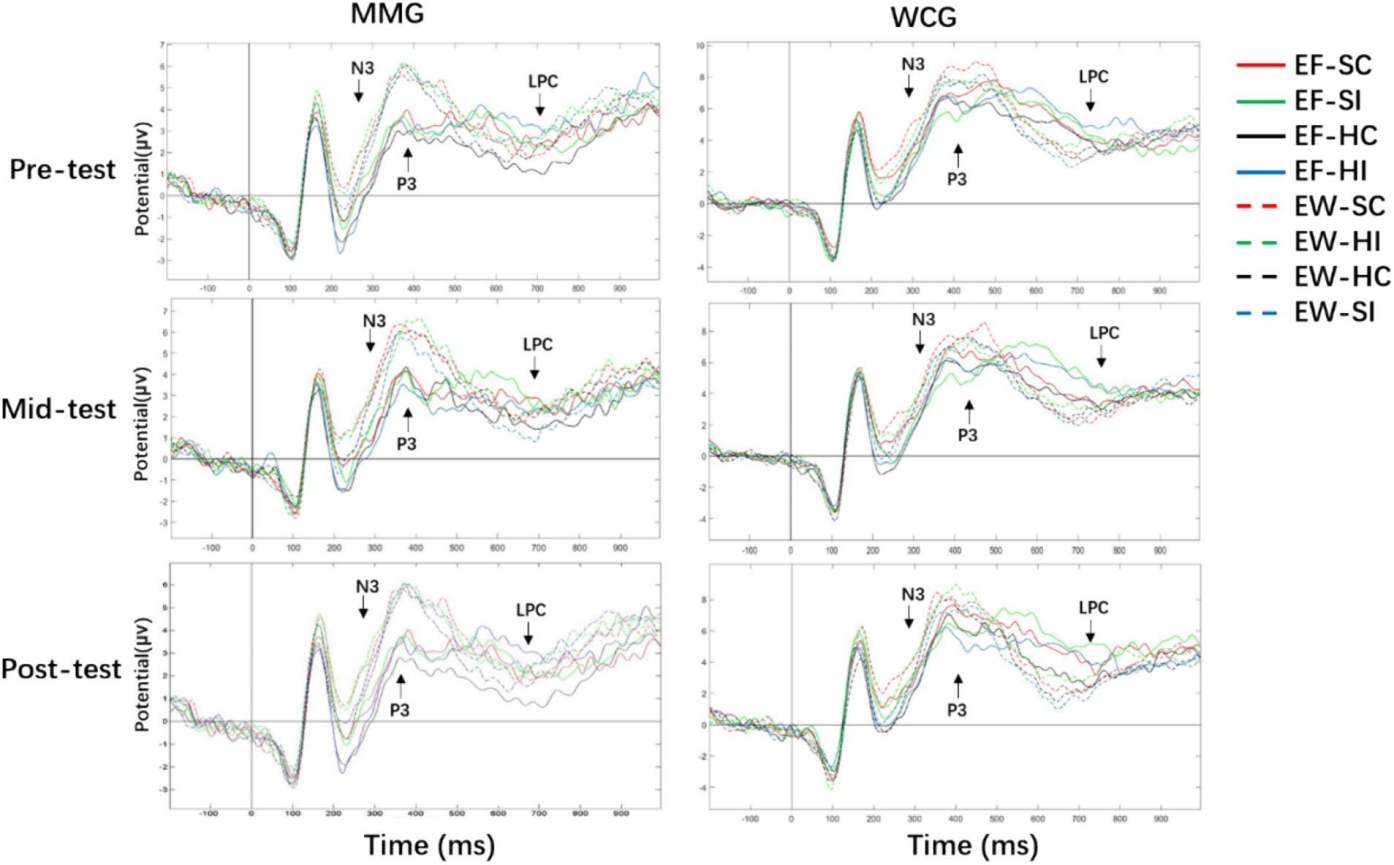
Grand average waveforms of N3, P3, and late positive component (LPC) at electrode CPz in three test of the emotional face–word Stroop task. MMG, mindfulness-based music listening group; WCG, wait-list control group; EF-SC, emotional faces-sad congruency; EF-SI, emotional faces-sad incongruency; EF-HC, emotional faces-happy congruency; EF-HI, emotional faces-happy incongruency; EW-SC, emotional words-sad congruency; EW-HI, emotional words-happy incongruency; EW-HC, emotional words-happy congruency; EW-SI, emotional words-sad incongruency.

The ERP results for the emotional face-word Stroop task under the three mood states are presented in Table 4 and Figure 5.

**Table 4:** Descriptive statistics of the ERP results in three tests of emotional face-word Stroop task.

Variables			MMG (M ± SD)					WCG (M ± SD)						
			*n* = 30					*n* = 30						
			Pre-test		Mid-test		Post-test		Pre-test		Mid-test		Post-test	
N3	EFs	SC	1.20	0.58	1.34	0.58	0.58	0.64	2.48	0.58	1.68	0.58	2.28	0.64
		SI	0.68	0.63	1.57	0.52	0.85	0.63	1.99	0.63	1.28	0.52	2.22	0.63
		HC	0.89	0.57	0.93	0.56	−0.08	0.68	1.97	0.57	1.04	0.56	1.54	0.68
		HI	0.83	0.58	0.72	0.61	0.36	0.62	1.54	0.58	1.33	0.61	1.77	0.62
	EWs	SC	1.69	0.58	3.13	0.67	2.64	0.73	3.62	0.58	2.49	0.67	3.31	0.73
		SI	1.94	0.58	2.77	0.57	2.46	0.65	3.05	0.58	2.27	0.57	3.03	0.65
		HC	1.70	0.49	2.69	0.55	1.96	0.64	2.58	0.49	2.12	0.55	2.43	0.64
		HI	1.09	0.59	1.91	0.57	1.90	0.68	2.63	0.59	1.83	0.57	2.18	0.68
P3	EFs	SC	3.28	0.53	2.55	0.64	2.31	0.63	4.83	0.53	4.37	0.64	5.02	0.63
		SI	2.50	0.55	2.28	0.58	1.97	0.65	3.70	0.55	3.06	0.58	3.95	0.65
		HC	2.92	0.52	2.32	0.57	1.38	0.65	4.28	0.52	3.57	0.57	4.23	0.65
		HI	2.78	0.53	1.75	0.59	1.91	0.66	4.00	0.53	3.62	0.59	3.71	0.66
	EWs	SC	3.72	0.59	4.46	0.65	4.34	0.68	6.00	0.59	5.04	0.65	5.54	0.68
		SI	3.92	0.56	4.61	0.59	4.12	0.70	5.21	0.56	4.39	0.59	5.58	0.70
		HC	3.94	0.54	4.37	0.61	3.84	0.69	5.25	0.54	4.69	0.61	4.93	0.69
		HI	3.93	0.61	3.91	0.61	4.19	0.70	5.21	0.61	4.40	0.61	4.82	0.70
LPC	EFs	SC	3.09	0.53	2.22	0.40	2.11	0.48	3.32	0.53	3.05	0.46	3.66	0.42
		SI	2.65	0.46	2.21	0.46	2.22	0.53	3.17	0.46	3.48	0.51	3.88	0.49
		HC	2.23	0.44	1.45	0.45	1.44	0.52	3.08	0.44	2.57	0.46	2.81	0.51
		HI	2.61	0.49	2.56	0.44	1.69	0.54	3.77	0.49	3.32	0.50	3.14	0.49
	EWs	SC	2.31	0.38	2.67	0.48	2.29	0.45	3.36	0.38	2.49	0.46	2.73	0.47
		SI	2.38	0.38	2.66	0.43	2.47	0.43	3.53	0.38	2.89	0.42	2.90	0.44
		HC	2.82	0.50	2.44	0.42	2.23	0.42	3.15	0.50	2.61	0.38	2.37	0.45
		HI	2.81	0.44	2.81	0.45	1.37	0.53	2.76	0.44	2.72	0.42	1.95	0.55

EFs, emotional faces; EWs, emotional words; SC, sad congruency; SI, sad incongruency; HC, happy congruency; HI, happy incongruency; MMG, mindfulness-based music listening group; WCG, wait-list control group; M, mean; SD, standard deviation.

#### 3.3.1 N3

The main effects of the test and group were not recorded. There was a main effect of electrodes on N3 (*F* (5, 54) = 46.96, *p* < 0.001, *η*2 *p* = 0.45), and the post-hoc *t*-test suggested that N3 mean amplitudes of FZ and OZ were greater than those of other electrodes (*p* < 0.05), with ratings of N3 mean amplitudes in Oz > Fz > FCz > Cz > CPz > Pz. There was a main effect of the condition (*F* (3, 56) = 12.26, *p* < 0.001, *η*2 *p* = 0.40), and N3 mean amplitudes of sad congruency and incongruency were greater than those of happy congruency and incongruency, with no differences between sad congruency and incongruency or happy congruency and incongruency. An interaction of the group and electrode was observed (*F* (5, 58) = 2.94, *p* = 0.013, *η*2 *p* = 0.09), and N3 mean amplitudes for MMG were greater than those for WCG in FZ (*p* = 0.019), with no significant differences in five other electrodes. There was a triple interaction effect of the test, electrode, and group (*F* (1, 58) = 2.36, *p* = 0.01, *η*2 *p* = 0.04), simple effect analysis indicated that N3 mean amplitudes of the MMG were greater than those of the WCG on FZ in pre- and post-test (*p* < 0.01), and N3 mean amplitudes of the MMG were greater than that of the WCG on Oz in post-test (*p* = 0.023); there were no between-group differences in mid-test (*p* > 0.05). No quadruple interaction effect between the test, condition, electrode, and group was recorded.

#### 3.3.2 P3

There were no main effects of the test. A main effect of the group was recorded (*F* (1, 58) = 4.50, *p* = 0.038, *η*2 *p* = 0.07), and the P3 mean amplitudes for MMG were smaller than that for WCG. There was a main effect of the electrodes (*F* (5, 54) = 59.88, *p* < 0.001, *η*2 *p* = 0.51), and the P3 mean amplitudes of CPz were greater than those of five other electrodes (*p* < 0.001), with ratings of the P3 mean amplitudes in CPz > Pz > Cz > FCz > Fz > Oz. There was a main effect of the condition (*F* (3, 56) = 11.10, *p* < 0.001, *η*2 *p* = 0.37), and the P3 mean amplitudes of sad congruency were greater than those of three other conditions (*p* = 0.016), with no significant differences between three other conditions. There was an interaction of the test and group (*F* (1, 58) = 5.11, *p* = 0.018, *η*2 *p* = 0.08), and simple analysis effect showed that the P3 mean amplitudes for MMG were smaller than those for WCG in pre- and post-test (*p* = 0.038), with no significant between-group difference in mid-test. There was an interaction of the electrode and group (*F* (5, 58) = 4.31, *p* = 0.014, *η*2 p = 0.07), the P3 mean amplitudes of the MMG were smaller than those of the WCG in Fz, Cz, and CPz (*p* = 0.042), there were no significant between-group differences in FCz, Pz, and Oz. A triple interaction effect of test, electrode, and group was observed (*F* (2, 58) = 2.70, *p* = 0.003, *η*2 *p* = 0.05), and the P3 mean amplitudes for MMG were smaller than those for WCG at Fz and CPz (*p* < 0.01), with no between-group differences at the other four electrodes in the pre-test. There were no significant between-group differences on all electrodes in the mid-test. In the post-test phase, except for the Pz, the P3 amplitudes of the five electrodes in the MMG were smaller than those in the WCG. There was no quadruple interaction effect between the test, condition, electrode, and group.

#### 3.3.3 LPC

No significant effects of the tests were observed. The main effect of the group was marginally significant (*F* (1, 58) = 3.76, *p* = 0.057, *η*2 *p* = 0.06), and LPC mean amplitudes in the MMG were smaller than that in the WCG. A main effect of the electrode (*F* (5, 58) = 159.08, *p* < 0.001, *η*2 *p* = 0.73) was found, LPC mean amplitudes of Fz, FCz, Cz, and CPz were greater than those of PZ and OZ (*p* < 0.01), and there were no significant differences between Fz, FCz, Cz, and CPz. The main effect of the condition was observed (*F* (3, 58) = 3.94, *p* = 0.009, *η*2 p = 0.06), and LPC mean amplitudes of sad congruency and incongruency were greater than those of happy congruency, with no difference between sad congruency and incongruency or happy congruency and incongruency. An interaction of the test and group was marginally significant (*F* (1, 58) = 3.42, *p* = 0.061, *η*2 p = 0.05), and LPC mean amplitudes for MMG were smaller than that for WCG in the post-test, with no significant between-group differences in pre- and mid-test. There was an interaction of the electrode and group (*F* (5, 58) = 3.78, *p* = 0.002, *η*2 p = 0.06), LPC mean amplitudes for MMG were smaller than those for WCG in Fz, Cz, and CPz (*p* < 0.05), and no significant differences between-group were showed in FCz, Pz, and Oz. A triple interaction effect of the test, electrode, and group was recorded (*F* (2, 58) = 1.95, *p* = 0.036, *η*2 p = 0.03), and the mean LPC amplitudes for MMG were smaller than those for WCG on Fz, FCz, Cz, and CPz in the mid-test and on Cz, CPz, and Oz in the post-test; the differences between groups in the pre-test were not observed. No other triple or quadruple interaction effects of the test, condition, electrode, and group were observed.

## 4 Discussion

In the current study, conflict control in young adults with insomnia disorders was investigated using questionnaire-Stroop task-ERP technology. The research results found that compared to the WCG, the MMG showed higher ACC and slower RTs and induced greater N3 amplitude and smaller P3 and LPC amplitudes in the post-test phase, indicating MBML intervention effectively improved the conflict control and attentional processing in young adults with ID. In the mid-test phase, the negative emotions induced by the negative simulated video significantly suppressed attention processing in conflict control of young adults with ID. The results of the current study indicate that consistent with existing research results (Blasco-Magraner et al., 2023; Liu et al., 2021a,c; Luo et al., 2024; Liu et al., 2020a,b), different emotional states have an impact on conflict control in young adults with ID (Ding et al., 2023; Ding et al., 2024; Dressle and Riemann, 2023). The crucial implications of this study lie in the fact that the MBML strategy provides a new intervention pathway for individuals with ID, such as impaired conflict control. Importantly, this study also provides a theoretical supplement for the clinical treatment of insomniacs’ hyperarousal and conflict control.

### 4.1 MBML intervention improves some aspects of conflict control and attentional processing in individuals with ID

Consistent with existing studies (Liu et al., 2019b, 2021a,c; Luo et al., 2024; Liu et al., 2020a,b), MBML intervention effectively regulated negative emotions induced by an 8-min simulated video and improved positive emotion among young adults with ID. The results of combining questionnaire tasks and ERP indicators suggest that the effective integration of mindfulness meditation and calm music significantly promotes attention processing of conflict control (Ziegler et al., 2019) and cognitive function in young adults with ID (Ding et al., 2024; Liu et al., 2021c; Loo et al., 2020). Based on our questionnaire results and existing research findings (Marchand, 2012; Zhao et al., 2021), MBML reduced levels of cognitive and somatic arousal in individuals with ID.

In the emotional-face sub-task, the Stroop effect on accuracy and response time was significant. Negative emotional states induced by the 8-min simulated video inhibited the processing of happy or sad faces in conditions of congruency or incongruency (lower ACC and slower RTs), whereas the MBML intervention effectively promoted the processing of happy or sad faces in conditions of congruency or incongruency (higher ACC and faster RTs) among young adults with ID. In the emotional word sub-task, there was no significant between-group difference in ACC, but there was a significant Stroop effect on RTs. In the pre-test phase, the RTs of the MMG were faster than those of the WCG, which may be a cue to the higher positive emotions of the MMG compared to those of the WCG (see Table 1). However, there were no significant between-group differences in the negative emotion induction phase of the mid-test. In the intervention phase of the post-test, the MBML improved the RTs of young adults with ID for emotional words. Our results are consistent with those of previous studies (Ding et al., 2023; Liu et al., 2021a; Luo et al., 2024; Liu et al., 2020a,b) in that negative emotions have an inhibitory effect on individual conflict control and semantic processing.

In terms of conflict control performance in the emotional face-word Stroop task, compared with the pre-test (baseline phase), lower ACC and slower RTs were found during the emotional faces sub-task, and slower RTs were recorded in the emotional words sub-task in the mid-test (negative emotion induction phase). In the post-test (MBML intervention phase), higher ACC and faster RTs were observed in the emotional faces sub-task, and faster RTs were found in the emotional word sub-task. In the emotion-cognition interaction, our task and questionnaire results indicated that the negative emotions induced by the 8-minute simulated video had a negative impact on conflict control performance among young adults with ID. This indicates that negative emotions (upset, distressed, scared, terrified) exacerbate the impaired conflict control in young adults with ID. However, in the post-test phase, MBML intervention improved conflict control in young adults with ID, enhanced the levels of cognitive and somatic arousal, and induced positive emotions. Our ERP results in the emotional face-word Stroop task confirmed the behavioral performance in attention processing of conflict control for ID, exhibiting greater N3 effects and smaller P3 and LPC effects.

### 4.2 Electrophysiological mechanism of MBML intervention in mitigating conflict control for ID

Our ERP results indicated that the N3, P3, and LPC are EEG indicators that reflect the impact of emotional states on conflict control. Compared to the pre-test phase, although the amplitudes of N3, P3, and LPC showed no statistically significant differences, the P3 and LPC amplitudes decreased for all participants in the mid-test phase. In the intervention phase of the post-test, compared with the WCG, the MBML intervention induced a larger N3 effect in the frontal region and a smaller N3 effect in the occipital region. This indicates that N3, as an EEG indicator reflecting an individual’s emotional state, shows smaller effects with positive emotions and larger effects with negative emotions (Draschkow et al., 2018; Liu et al., 2021a; Maguire et al., 2013; Truman and Mudrik, 2018).

Our ERP results indicate that negative emotions induce smaller P3 effects, whereas larger P3 effects are associated with positive emotions. In the post-test intervention phase, MBML significantly reduced the P3 amplitude in five regions (frontal, frontal-central, central, central–parietal, and occipital regions), which is consistent with previous studies (Ding et al., 2023; Liu et al., 2020a,b, 2021a). Our ERP results reveal that the P3 and LPC are effective indicators for detecting MBML intervention strategies, which can effectively evaluate the conflict control performance of individuals with ID in emotion–cognition interactions. Compared to the WCG, the smaller LPC effect indicates that MBML effectively regulates negative emotions and promotes conflict control in individuals with ID.

Compared to the WCG, the smaller LPC effect indicates that MBML promotes their attention processing of conflict control in the post-test intervention phase. Studies have shown that LPC is associated with higher cognitive resources (Liu et al., 2020a,b) and that listening to calm music in a state of mindfulness can effectively reduce the consumption of higher cognitive resources and alleviate individual cognitive loads (Guo and Koelsch, 2015; Liu et al., 2021a). Based on these advantages in the emotion–cognition interaction, our ERP results support the positive role of MBML intervention strategies in regulating negative emotions among young adults with ID and improving facial and semantic processing in conflict control (Raschle et al., 2017; Zhu et al., 2010).

## 5 Conclusion

In summary, the findings of this study suggest that in comparison with other young adults with insomnia who did not engage in the MBML intervention, maintaining mindfulness while listening to music may enhance positive emotional experiences, improve cognitive ability, and exhibit larger N3 effects and smaller P3 and LPC effects in the electrophysiology mechanism. Compared to a negative emotional state, a positive mood state evoked by mindfulness-based music listening significantly regulated cognitive control and enhanced attentional processing in young Chinese adults with insomnia disorders. Negative mood induced by the simulated video suppressed the attention processing of conflict control in young adults with insomnia disorders, and mindfulness-based music listening effectively improves negative mood and enhances the attention processing of conflict control in the emotional face-word Stroop task.

However, it should be noted that there are some limitations. First, although the MBML could regulate negative emotions and reduce the level of hyperarousal in young Chinese adults with insomnia disorders, the conclusions of this study may not be generalizable to other age groups (such as adolescents or elderly populations) and non-Chinese participants. Second, the medium- to long-term intervention effects of the MBML were not systematically tested in different age groups with insomnia disorders. Third, although MBML could effectively improve the conflict control level of young adults with insomnia disorders, MBML intervention strategies should be optimized based on different music styles and music preferences, targeting different symptoms of insomnia disorders in future, such as difficulties in falling asleep, non-restorative sleep, and impaired daytime functioning (Carney et al., 2013; Riemann et al., 2010). Fourth, fMRI-EEG fusion technology could be used to reveal the neural mechanisms underlying the improvement in somatic and cognitive hyperarousal in young adults with insomnia disorders through medium- to long-term MBML intervention strategies.

To our knowledge, this is the first study to explore the effect of mindfulness-based music listening on the attentional processing of conflict control among young adults with insomnia disorders and provide new intervention methods for the future studies on the integration of music therapy with other psychological therapies among young adults with other sleep disorders.

## Data Availability

The original contributions presented in the study are included in the article/supplementary material, further inquiries can be directed to the corresponding authors.

## References

[ref1] Blasco-Magraner J. S. Bernabe-Valero G. Marin-Liebana P. Botella-Nicolas A. M. (2023). Changing positive and negative affects through music experiences: a study with university students. BMC Psychol 11:76. doi: 10.1186/s40359-023-01110-9 36944996 PMC10031901

[ref2] Cao X.-L. Wang S.-B. Zhong B.-L. Zhang L. Ungvari G. S. Ng C. H.. (2017). The prevalence of insomnia in the general population in China: a meta-analysis. PLoS One 12:e0170772. doi: 10.1371/journal.pone.0170772, PMID: 28234940 PMC5325204

[ref3] Carlson L. E. Brown K. W. (2005). Validation of the mindful attention awareness scale in a cancer population. J. Psychosom. Res. 58, 29–33. doi: 10.1016/j.jpsychores.2004.04.366 15771867

[ref4] Carney C. E. Harris A. L. Falco A. Edinger J. D. (2013). The relation between insomnia symptoms, mood, and rumination about insomnia symptoms. J. Clin. Sleep Med. 9, 567–575. doi: 10.5664/jcsm.2752 23772190 PMC3659377

[ref5] Carretié L. Iglesias J. García T. Ballesteros M. (1997). N300, P300 and the emotional processing of visual stimuli. Electroencephalogr. Clin. Neurophysiol. 103, 298–303. doi: 10.1016/S0013-4694(96)96565-7 9277632

[ref6] Chang E.-T. Lai H.-L. Chen P.-W. Hsieh Y.-M. Lee L.-H. (2012). The effects of music on the sleep quality of adults with chronic insomnia using evidence from polysomnographic and self-reported analysis: a randomized control trial. Int. J. Nurs. Stud. 49, 921–930. doi: 10.1016/j.ijnurstu.2012.02.019 22494532

[ref7] Chin T. Rickard N. S. (2013). Emotion regulation strategy mediates both positive and negative relationships between music uses and well-being. Psychol. Music 42, 692–713. doi: 10.1177/0305735613489916

[ref8] Chung P.-K. Zhang C.-Q. (2014). Psychometric validation of the Toronto mindfulness scale – trait version in Chinese college students. Eur. J. Psychol. 10, 726–739. doi: 10.5964/ejop.v10i4.776

[ref9] Deng Y.-Q. Li S. Tang Y.-Y. Zhu L.-H. Ryan R. Brown K. (2012). Psychometric properties of the Chinese translation of the mindful attention awareness scale (MAAS). Mindfulness 3, 10–14. doi: 10.1007/s12671-011-0074-1

[ref10] Dennis T. A. (2010). Neurophysiological markers for child emotion regulation from the perspective of emotion-cognition integration: current directions and future challenges. Dev. Neuropsychol. 35, 212–230. doi: 10.1080/87565640903526579 20390603 PMC2856094

[ref11] Dickson G. T. Schubert E. (2019). How does music aid sleep? Literature review. Sleep Med. 63, 142–150. doi: 10.1016/j.sleep.2019.05.016 31655374

[ref12] Ding X. He L. Geng X. Zhao X. He Z. Zhang X. (2023). Altered electrophysiology mechanism related to inhibitory control in adults with insomnia. Front. Neurol. 14:1271264. doi: 10.3389/fneur.2023.1271264 38073615 PMC10702741

[ref13] Ding X. He L. Kang T. Yang Y. Ji H. Zhao H.. (2024). The role of the left dorsolateral prefrontal cortex in conflict control during insomnia disorder. J. Psychiatr. Res. 171, 271–276. doi: 10.1016/j.jpsychires.2024.02.010 38330626

[ref14] Dopheide J. A. (2020). Insomnia overview: epidemiology, pathophysiology, diagnosis and monitoring, and nonpharmacologic therapy. Am. J. Manag. Care 26, S76–S84. doi: 10.37765/ajmc.2020.42769 32282177

[ref15] Draschkow D. Heikel E. Võ M. L.-H. Fiebach C. J. Sassenhagen J. (2018). No evidence from MVPA for different processes underlying the N300 and N400 incongruity effects in object-scene processing. Neuropsychologia 120, 9–17. doi: 10.1016/j.neuropsychologia.2018.09.016 30261162

[ref16] Dressle R. J. Riemann D. (2023). Hyperarousal in insomnia disorder: current evidence and potential mechanisms. J. Sleep Res. 32:e13928. doi: 10.1111/jsr.13928 37183177

[ref17] Edinger J. D. Arnedt J. T. Bertisch S. M. Carney C. E. Harrington J. J. Lichstein K. L.. (2021). Behavioral and psychological treatments for chronic insomnia disorder in adults: an American Academy of sleep medicine clinical practice guideline. J. Clin. Sleep Med. 17, 255–262. doi: 10.5664/jcsm.8986 33164742 PMC7853203

[ref18] Gong H. Ni C.-X. Liu Y.-Z. Zhang Y. Su W.-J. Lian Y.-J.. (2016). Mindfulness meditation for insomnia: a meta-analysis of randomized controlled trials. J. Psychosom. Res. 89, 1–6. doi: 10.1016/j.jpsychores.2016.07.016 27663102

[ref19] Guo S. Koelsch S. (2015). The effects of supervised learning on event-related potential correlates of music-syntactic processing. Brain Res. 1626, 232–246. doi: 10.1016/j.brainres.2015.01.046 25660849

[ref20] Hernandez-Ruiz E. Dvorak A. L. (2020). Music and mindfulness meditation: comparing four music stimuli composed under similar principles. Psychol. Music 49, 1–17. doi: 10.1177/0305735620969798

[ref21] Huang C.-Y. Chang E.-T. Hsieh Y.-M. Lai H.-L. (2017). Effects of music and music video interventions on sleep quality: a randomized controlled trial in adults with sleep disturbances. Complement. Ther. Med. 34, 116–122. doi: 10.1016/j.ctim.2017.08.015 28917363

[ref22] Ireland M. J. Day J. J. Clough B. A. (2019). Exploring scale validity and measurement invariance of the Toronto mindfulness scale across levels of meditation experience and proficiency. J. Clin. Psychol. 75, 445–461. doi: 10.1002/jclp.22709 30431146

[ref23] Lai H.-L. Chang E.-T. Li Y.-M. Huang C.-Y. Lee L.-H. Wang H.-M. (2014). Effects of music videos on sleep quality in middle-aged and older adults with chronic insomnia. Biol. Res. Nurs. 17, 340–347. doi: 10.1177/1099800414549237 25237150

[ref24] Lau M. A. Bishop S. R. Segal Z. V. Buis T. Anderson N. D. Carlson L.. (2006). The Toronto mindfulness scale: development and validation. J. Clin. Psychol. 62, 1445–1467. doi: 10.1002/jclp.20326 17019673

[ref25] Liu Y. Gao X. Zhao J. Zhang L. Chen H. (2020a). Neurocognitive correlates of food-related response inhibition in overweight/obese adults. Brain Topogr. 33, 101–111. doi: 10.1007/s10548-019-00730-y 31564028

[ref26] Liu X. Liu Y. Shi H. Li L. Zheng M. (2021a). Regulation of mindfulness-based music listening on negative emotions related to COVID-19: an ERP study. Int. J. Environ. Res. Public Health 18:7063. doi: 10.3390/ijerph18137063 34280999 PMC8296951

[ref27] Liu X. Liu Y. Shi H. Zheng M. (2021b). Effects of mindfulness meditation on musical aesthetic emotion processing. Front. Psychol. 12, –648062. doi: 10.3389/fpsyg.2021.648062 PMC8334183 34366968

[ref28] Liu Y. Liu X. Zheng M. (2023). A correlation study of music training, adult attachment, and personality traits using a large-sample questionnaire. Front. Psychol. 14:1218848. doi: 10.3389/fpsyg.2023.1218848 37691808 PMC10484518

[ref29] Liu Y. Quan H. Song S. Zhang X. Chen H. (2019a). Decreased conflict control in overweight Chinese females: Behavioral and event-related potentials evidence. Nutrients 11:1450. doi: 10.3390/nu11071450 31252512 PMC6683057

[ref30] Liu X. Shi H. Liu Y. Yuan H. Zheng M. (2021c). Mindfulness meditation improves musical aesthetic emotion processing in young adults. Int. J. Environ. Res. Public Health 18:13045. doi: 10.3390/ijerph182413045 34948651 PMC8701887

[ref31] Liu Y. Zhang L. Jackson T. Wang J. Yang R. Chen H. (2020b). Effects of negative mood state on event-related potentials of restrained eating subgroups during an inhibitory control task. Behav. Brain Res. 377:112249. doi: 10.1016/j.bbr.2019.112249 31541673

[ref32] Liu Y. Zhao J. Zhang X. Gao X. Xu W. Chen H. (2019b). Overweight adults are more impulsive than normal weight adults: evidence from ERPs during a chocolate-related delayed discounting task. Neuropsychologia 133:107181. doi: 10.1016/j.neuropsychologia.2019.107181 31476320

[ref33] Loo L.-M. Prince J. B. Correia H. M. (2020). Exploring mindfulness attentional skills acquisition, psychological and physiological functioning and wellbeing: using mindful breathing or mindful listening in a NonClinical sample. Psychomusicology: Music, Mind, and Brain 30, 103–118. doi: 10.1037/pmu0000255

[ref34] Luo X. Zhang A. Li H. Li Y. Ying F. Wang X.. (2024). The role of arts therapies in mitigating sleep initiation and maintenance disorders: a systematic review. Front. Psych. 15:1386529. doi: 10.3389/fpsyt.2024.1386529 PMC11137235 38818021

[ref35] Madsen J. Margulis E. H. Simchy-Gross R. Parra L. C. (2019). Music synchronizes brainwaves across listeners with strong effects of repetition, familiarity and training. Sci. Rep. 9:3576. doi: 10.1038/s41598-019-40254-w 30837633 PMC6401073

[ref36] Maguire M. J. Magnon G. Ogiela D. A. Egbert R. Sides L. (2013). The N300 ERP component reveals developmental changes in object and action identification. Dev. Cogn. Neurosci. 5, 1–9. doi: 10.1016/j.dcn.2012.11.008 23287022 PMC6987807

[ref37] Majeed N. M. Lua V. Y. Q. Chong J. S. Lew Z. Hartanto A. (2021). Does bedtime music listening improve subjective sleep quality and next-morning well-being in young adults? A randomized cross-over trial. Psychomusicology: Music, Mind, and Brain 31, 149–158. doi: 10.1037/pmu0000283

[ref38] Marchand W. R. (2012). Mindfulness-based stress reduction, mindfulness-based cognitive therapy, and zen meditation for depression, anxiety, pain, and psychological distress. J. Psychiatr. Pract. 18, 233–252. doi: 10.1097/01.pra.0000416014.53215.86 22805898

[ref39] Mengfan L. Guang Y. Guizhi X. (2020). The effect of the graphic structures of humanoid robot on N200 and P300 potentials. IEEE transactions on neural systems and rehabilitation engineering: a publication of the IEEE Engineering in Medicine and Biology Society. 10.1109/TNSRE.2020.3010250 32746323

[ref40] Miranda D. (2021). Neuroticism, musical emotion regulation, and mental health. Psychomusicology 31, 59–73. doi: 10.1037/pmu0000275

[ref41] Mizrahi Lakan S. Millgram Y. Tamir M. (2023). Desired sadness, happiness, fear and calmness in depression: the potential roles of valence and arousal. Emotion 23, 1130–1140. doi: 10.1037/emo0001120 35951386

[ref42] Nicassio P. M. Mendlowitz D. R. Fussell J. J. Petras L. (1985). The phenomenology of the pre-sleep state: the development of the pre-sleep arousal scale. Behav. Res. Ther. 23, 263–271. doi: 10.1016/0005-7967(85)90004-X 4004706

[ref43] Raschle N. M. Fehlbaum L. V. Menks W. M. Euler F. Sterzer P. Stadler C. (2017). Investigating the neural correlates of emotion-cognition interaction using an affective Stroop task. Front. Psychol. 8:1489. doi: 10.3389/fpsyg.2017.01489 28919871 PMC5585191

[ref44] Riemann D. Spiegelhalder K. Feige B. Voderholzer U. Berger M. Perlis M.. (2010). The hyperarousal model of insomnia: a review of the concept and its evidence. Sleep Med. Rev. 14, 19–31. doi: 10.1016/j.smrv.2009.04.002 19481481

[ref45] Rusch H. L. Rosario M. Levison L. M. Olivera A. Livingston W. S. Wu T.. (2018). The effect of mindfulness meditation on sleep quality: a systematic review and meta-analysis of randomized controlled trials. Ann. N. Y. Acad. Sci. 1445, 5–16. doi: 10.1111/nyas.13996 30575050 PMC6557693

[ref46] Schneider M. N. Denis D. Buysse D. J. Kovas Y. Gregory A. M. (2019). Associations between pre-sleep arousal and insomnia symptoms in early adulthood: a twin and sibling study. Sleep 42:zsz029. doi: 10.1093/sleep/zsz029 30722021

[ref47] Si-yi C. Hong C. Ren-lai Z. Yan-yan J. (2012). Revision of mindful attention awareness scale (MAAS). Chin. J. Clin. Psych. 20, 148–151. doi: 10.16128/j.cnki.1005-3611.2012.02.024

[ref48] Smith T. Panfil K. Bailey C. Kirkpatrick K. (2019). Cognitive and behavioral training interventions to promote self-control. J Exp Psychol Anim Learn Cogn 45, 259–279. doi: 10.1037/xan0000208 31070430 PMC6716382

[ref49] Susino M. (2023). Emotional expression, perception, and induction in music and dance: considering ecologically valid intentions. J. Creat. Behav. doi: 10.1002/jocb.587

[ref50] Tomaselli K. A. (2014). The effect of mindfulness-based music listening on the anxiety symptoms and awareness of older adults in a senior living facility. Tallahassee, FL: Florida State University.

[ref51] Truman A. Mudrik L. (2018). Are incongruent objects harder to identify? The functional significance of the N300 component. Neuropsychologia 117, 222–232. doi: 10.1016/j.neuropsychologia.2018.06.004 29885960

[ref52] Uddin L. Q. (2021). Cognitive and behavioural flexibility: neural mechanisms and clinical considerations. Nat. Rev. Neurosci. 22, 167–179. doi: 10.1038/s41583-021-00428-w 33536614 PMC7856857

[ref53] Umemoto A. Cole S. L. Allison G. O. Dolan S. Lazarov A. Auerbach R. P.. (2021). Neurophysiological predictors of gaze-contingent music reward therapy among adults with social anxiety disorder. J. Psychiatr. Res. 143, 155–162. doi: 10.1016/j.jpsychires.2021.09.022 34487992 PMC8557124

[ref54] Wang Y. Luo Y. (2005). Standardization and assessment of college Students' facial expression of emotion Chinese. J. Clin. Psychol. 13, 21–23. doi: 10.16128/j.cnki.1005-3611.2005.04.006

[ref55] Wardle-Pinkston S. Slavish D. C. Taylor D. J. (2019). Insomnia and cognitive performance: a systematic review and meta-analysis. Sleep Med. Rev. 48:101205. doi: 10.1016/j.smrv.2019.07.008 31522135

[ref56] Watson D. Clark L. A. (1988). Development and validation of brief measures of positive and negative affect: the PANAS scales. J. Pers. Soc. Psychol. 54, 1063–1070. doi: 10.1037//0022-3514.54.6.1063 3397865

[ref57] Weth K. Raab M. H. Carbon C.-C. (2015). Investigating emotional responses to self-selected sad music via self-report and automated facial analysis. Music. Sci. 19, 412–432. doi: 10.1177/1029864915606796

[ref58] Wittmann M. Schmidt S. (2014). “Mindfulness meditation and the experience of time” in Meditation – Neuroscientific approaches and philosophical implications. eds. Schmidt S. Walach H. (Springer International Publishing), 199–209.

[ref59] Xue S. Ren G. Kong X. Liu J. Qiu J. (2015). Electrophysiological correlates related to the conflict adaptation effect in an emotional conflict task. Neurosci. Lett. 584, 219–223. doi: 10.1016/j.neulet.2014.10.019 25459295

[ref60] Yuan J. Zhang Q. Chen A. Li H. Wang Q. Zhuang Z.. (2007). Are we sensitive to valence differences in emotionally negative stimuli? Electrophysiological evidence from an ERP study. Neuropsychologia 45, 2764–2771. doi: 10.1016/j.neuropsychologia.2007.04.018 17548095

[ref61] Zhao W. Gao D. Yue F. Wang Y. Mao D. Chen X.. (2018). Response inhibition deficits in insomnia disorder: an event-related potential study with the stop-signal task. Front. Neurol. 9:610. doi: 10.3389/fneur.2018.00610 30131753 PMC6090996

[ref62] Zhao W. Van Someren E. J. W. Li C. Chen X. Gui W. Tian Y.. (2021). EEG spectral analysis in insomnia disorder: a systematic review and meta-analysis. Sleep Med. Rev. 59:101457. doi: 10.1016/j.smrv.2021.101457 33607464

[ref63] Zhu X. Lazarov A. Dolan S. Bar-Haim Y. Dillon D. G. Pizzagalli D. A.. (2023). Resting state connectivity predictors of symptom change during gaze-contingent music reward therapy of social anxiety disorder. Psychol. Med. 53, 3115–3123. doi: 10.1017/S0033291721005171 35314008 PMC9612546

[ref64] Zhu X. R. Zhang H. J. Wu T. T. Luo W. B. Luo Y. J. (2010). Emotional conflict occurs at an early stage: evidence from the emotional face-word Stroop task. Neurosci. Lett. 478, 1–4. doi: 10.1016/j.neulet.2010.04.036 20417689

[ref65] Ziegler D. A. Simon A. J. Gallen C. L. Skinner S. Janowich J. R. Volponi J. J.. (2019). Closed-loop digital meditation improves sustained attention in young adults. Nat. Hum. Behav. 3, 746–757. doi: 10.1038/s41562-019-0611-9 31160812 PMC7534732

